# Whole Genome Sequencing and Characterization of Multidrug-Resistant (MDR) Bacterial Strains Isolated From a Norwegian University Campus Pond

**DOI:** 10.3389/fmicb.2020.01273

**Published:** 2020-06-17

**Authors:** Misti D. Finton, Roger Meisal, Davide Porcellato, Lin T. Brandal, Bjørn-Arne Lindstedt

**Affiliations:** ^1^Faculty of Chemistry, Biotechnology and Food Science, Norwegian University of Life Sciences, Ås, Norway; ^2^Department of Zoonotic, Food- and Waterborne Infections, Norwegian Institute of Public Health, Oslo, Norway

**Keywords:** extended-spectrum β-lactamase (ESBL), multidrug resistant, heteropathogenic *Escherichia coli*, whole genome sequencing, MinION

## Abstract

The presence of extended-spectrum β-lactamase (ESBL)-producing bacteria in environmental sources has been reported worldwide and constitutes a serious risk of community-acquired infections with limited treatment options. The current study aimed to explore the presence of these worrisome bacteria in a pond located at the Norwegian University of Life Sciences in Ås, Norway. A total of 98 bacterial isolates survived growth on selective chromogenic media and were identified by 16S rRNA Sanger sequencing. All strains were evaluated for the presence of the most commonly found β-lactamases and ESBLs in clinical settings (*bla*_CTX–M_ groups 1, 2, and 9, *bla*_CMY_, *bla*_SHV_, and *bla*_TEM_) and carbapenemases (*bla*_IMP_, *bla*_KPC_, *bla*_NDM_, *bla*_OXA_, *bla*_SFC1_, *bla*_VIM_) through multiplex PCR. A total of eight strains were determined to contain one or more genes of interest. Phenotypic resistance to 18 antimicrobial agents was assessed and isolates were subjected to whole genome sequencing through a combination of Oxford Nanopore’s MinION and Illumina’s MiSeq. Results revealed the presence of β-lactamase and ESBL-producing *Escherichia coli*, *Klebsiella pneumoniae*, *Stenotrophomonas maltophilia*, and a *Paraburkholderia* spp. Identified β-lactamases and ESBLs include *bla*_CTX–M_, *bla*_TEM_, *bla*_CMY_, *bla*_SHV_ and a possible *bla*_KPC_-like gene, with both documented and novel sequences established. In addition, two inducible β-lactamases were found, a class A β-lactamase (L1) and a cephalosporinase (L2). All strains were determined to be multidrug resistant and numerous resistance genes to non-β-lactams were observed. In conclusion, this study demonstrates that environmental sources are a potential reservoir of clinically relevant ESBL-producing bacteria that may pose a health risk to humans upon exposure.

## Introduction

Antibiotic resistant bacteria (ARB) is a global public health threat that jeopardizes the successful treatment of infectious disease (World Health Organization [WHO], 2014). Currently, the most widely used class of drugs in human and veterinary medicine are the β-lactams, including penicillins and 3rd generation cephalosporins (Bush and Bradford, 2016). Due to persistent exposure of bacterial strains to these antibiotics, extended-spectrum β-lactamases (ESBLs) have evolved that hydrolyze the β-lactam ring, rendering these vital antibiotics ineffective (Reeba et al., 2019). As a result, ESBLs have emerged as one of the most clinically significant resistance mechanisms associated with limited therapeutic options (Pitout et al., 2005). The predominant ESBL detected in clinical settings is derived from *bla*_CTX–M_ (Bora et al., 2014), which is most frequently identified in Enterobacteriaceae (Pitout et al., 2005). ESBL-producing bacteria also commonly exhibit co-resistance to many other classes of antibiotics, further impeding the successful treatment of bacterial infections (Chaudhary and Aggarwal, 2004).

Although ESBL-producing bacteria have traditionally been considered a clinical problem, their presence in the environment has gained attention as an exposure route to humans through food items, drinking water, and direct contact with water bodies. Previous studies have identified aquatic ecosystems as “hot spots” for microorganisms from a variety of sources as well as the acquisition, evolution, and dissemination of ARB (Baquero et al., 2008; Machado and Bordalo, 2014; Tokajian et al., 2018). In addition, the added selective pressures of antimicrobials, biocides, heavy metals, and disinfectants promote the selection of bacterial defense mechanisms that uphold their survival and spread (Baquero et al., 2008; Marti et al., 2014; Tokajian et al., 2018).

Previous studies have typically focused on the detection of specific types of ESBL-producers in the environment, namely Enterobacteriaceae (Jang et al., 2013; Zurfluh et al., 2013; Kittinger et al., 2016; Zarfel et al., 2017). However, ESBLs are commonly located on plasmids that are readily transferrable between a variety of bacterial species (Jacoby, 1997). Therefore, the current study aimed to explore the presence of ESBLs found in diverse types of bacteria located in a local environmental water source. We also wished to examine the presence of the most clinically-relevant β-lactamases. In addition, we investigated the possibility of carbapenemase-producing bacteria, which are resistant to the last resort antibiotics, the carbapenems. Furthermore, we assessed resistance determinants, virulence factors (VFs), and phylogenetics traits characteristic of pathogenicity through whole genome sequencing (WGS).

## Materials and Methods

### Sampling Sites and Collection

**FIGURE 1:**
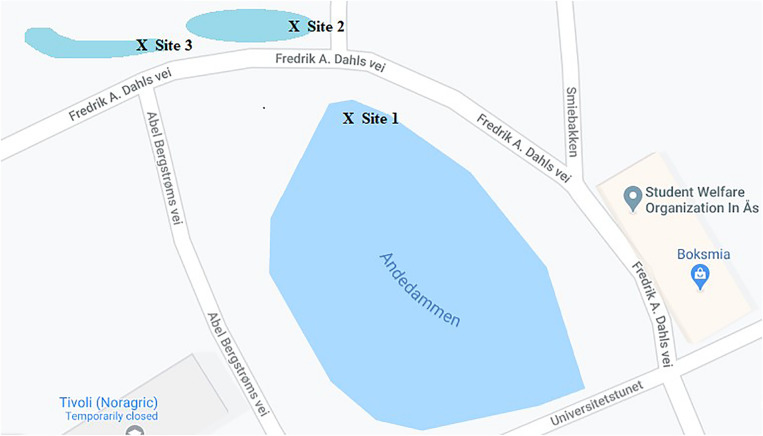
Map showing soil and water sampling sites selected on the NMBU campus where 98 water samples were collected for screening of ESBL and carbapenemase-producing bacterial isolates. Sample site 1 (59°40′02.6″N 10°46′09.0″E), sample site 2 (59°40′03.2″N 10°46′08.2″E), and sample site 3 (59°40′03.2″N 10°46′06.4″E).

Between August 2017 and January 2019, a total of 29 water samples were obtained from the campus pond (“Andedammen”) at the Norwegian University of Life Sciences (NMBU) in Ås, Norway (Figure 1). Samples were collected in sterile Pyrex^®^ wide mouth storage bottles. All samples were stored at 4°C until analysis.

Water quality testing was also performed using an EPA-approved commercial kit, Colilert, which simultaneously detects and quantifies both total coliforms and *E. coli* (IDEXX Laboratories, Inc., Maine, United States).

### Bacterial Isolation

Samples were coarsely filtered through Whatman^®^ Quantitative filter paper (589/1, black ribbon, 15 cm diameter). The filtered water from each site was divided into 100 ml portions in separate sterile glass bottles and vacuum-filtrated through EZ-Pak^®^ filters with a pore size of 0.45 μm (Merck, Darmstadt, Germany) on a Millipore Microfil Support Frit (Merck). The filter membranes were then transferred onto Brilliance^TM^ ESBL agar and Brilliance CRE^TM^ agar (Oxoid, Hampshire, United Kingdom) with a sterile tweezer and incubated for 24 h at 37°C. Per the manufacturer’s recommendations, plates that displayed weak growth were incubated for an additional 24 h and re-assessed. Single colonies with phenotypic differences were sub-cultured onto fresh plates and incubated at 37°C for approximately 24 h. To verify survival on the selective media, isolates were sub-cultured once more in the same manner.

### DNA Extraction and Identification

Genomic DNA was extracted with the GenElute Bacterial Genomic DNA Kit (Sigma-Aldrich, St. Louis, MI, United States). The kit’s elution buffer contains EDTA and was therefore substituted with the SequalPrep^TM^ Normalization Elution Buffer (Oxoid). All other manufacturer’s instructions were followed. DNA was assessed for purity and quantity with NanoDrop (Oxoid) and Qubit^®^ 2.0 (Life Technologies, Grand Island, NY, United States), respectively.

Species identification was determined through amplification of the 16S rRNA gene using the primers listed in Table 1. The reaction was conducted with 0.02 U/μl, iProof High-Fidelity DNA Polymerase (Bio-Rad Laboratories Inc., Hercules, CA, United States), 1X iProof Buffer, 0.25 μM of each primer, 200 nM dNTPs (Oxoid), and sterile H_2_O. All PCR reactions were performed for 35 cycles in a 40 μl final reaction mixture. The amplification conditions were as follows: 98°C for 30 s, followed by 35 cycles of 98°C for 15 s, 53°C for 30 s, 72°C for 20 s, followed by 72°C for 10 min.

**TABLE 1:** Primers used for species identification (16S) and the screening of β-lactamase, ESBL, and carbapenemase genes.

**Target genes**	**Primer Sequence (5**′**-3**′)	**Amplicon Size (bp)**	**References**
**Multiplex 1**			
blaOXA-48	F- GCTTGATCGCCCTCGATT	281	Dallenne et al., 2010
	R- GATTTGCTCCGTGGCCGAAA		
blaCTX-M (gr. 2)	F- CGTTAACGGCACGATGAC	404	Dallenne et al., 2010
	R- CGATATCGTTGGTGGTTCCAT		
blaOXA	F- GGCACCAGATTCAACTTTCAAG	564	Dallenne et al., 2010
	R- GACCCCAAGTTTCCTGTAAGTG		
blaSHV	F- AGCCGCTTGAGCAAATTAAAC	713	Dallenne et al., 2010
	R- ATCCCGCAGATAAATCACCAC		
**Multiplex 2**			
blaCTX-M (gr. 9)	F- TCAAGCCTGCCGATCTGGT	561	Dallenne et al., 2010
	R- TGATTCTCGCCGCTGAAG		
blaCTX-M (gr. 1)	F- TTAGGAARTGTGCCGCTGYA	688	Dallenne et al., 2010
	R- CGATATCGTTGGTGGTRCCAT		
blaTEM	F- CATTTCCGTGTCGCCCTTATTC	800	Dallenne et al., 2010
	R- CGTTCATCCATAGTTGCCTGAC		
**Multiplex 3**			
blaNDM	F- TGGCCCGCTCAAGGTATTTT	157	This study
	R- GTAGTGCTCAGTGTCGGCAT		
blaVIM	F- ATAGAGCACACTCGCAGACG	564	This study
	R- TTATTGGTCTATTTGACCGCGT		
blaKPC	F- TCCGTTACGGCAAAAATGCG	460	This study
	R- GCATAGTCATTTGCCGTGCC		
**Multiplex 4**			
*rpoB*	F- CAGGTCGTCACACGGTAACAAG	512	Universal primers
	R- GTGGTTCAGTTTCAGCATGTAC		
16S rRNA	F- AGAGTTTGATCMTGGCTCAG	1505	Universal primers
	R- GYTACCTTGTTACGACTT		
**Multiplex 5**			
*bla*_CMY_	F- GCATCTCCCAGCCTAATCCC	188	This study
	R- TTCTCCGGGACAACTTGACG		
*bla*_OXA–48_	F- GCTTGATCGCCCTCGATT	281	Dallenne et al., 2010
	R- GATTTGCTCCGTGGCCGAAA		
*bla*_IMP_	F- ACAGGGGGAATAGAGTGGCT	393	This study
	R- AGCCTGTTCCCATGTACGTT		
*bla*_VIM_	F- ATAGAGCACACTCGCAGACG	564	This study
	R- TTATTGGTCTATTTGACCGCGT		
**Multiplex 6**			
*bla*_NDM_	F- TGGCCCGCTCAAGGTATTTT	157	This study
	R- GTAGTGCTCAGTGTCGGCAT		
*bla*_SFC_	F- GGAGGGCGAATTGGGGTTTA	268	This study
	R- CACTGTACTGCAGAGTGGCA		
*bla*_KPC_	F- TCCGTTACGGCAAAAATGCG	460	This study
	R- GCATAGTCATTTGCCGTGCC		

### Detection of β-Lactamase Genes

All isolates were screened for genes that confer resistance to β-lactams (*bla*_CTX–M_ groups 1, 2, and 9, *bla*_CMY_, *bla*_SHV_, and *bla*_TEM_) and carbapenems (*bla*_IMP_, *bla*_KPC_, *bla*_NDM_, *bla*_OXA_, *bla*_SFC1_, and *bla*_VIM_) via multiplex PCR (Table 1) using DNA purified as described above. DNA amplification was performed in an Applied Biosystems^TM^ SimpliAmp Thermal Cycler (Applied Biosystems, Foster City, MA, United States) in a 25 μL final reaction mixture and amplified products were analyzed on a 1% agarose gel at 80 V-cm for 80 min. For all positive results, the process was repeated with individual primer pairs in separate reactions. The amplification conditions were as follows: 95°C for 15 min, followed by 28 cycles (Multiplex 1–4) or 30 cycles (Multiplex 5 and 6) of 94°C for 30 s, 60°C (Multiplex 5 and 6) or 62°C (Multiplex 1–4) for 90 s, 72°C for 90 s and 72°C for 10 min.

All positive products were purified with the GenElute^TM^ PCR Clean-up Kit (Sigma-Aldrich) and quantified with Qubit 2.0 (Life Technologies) following manufacturer’s instructions. Sanger sequencing was performed by GATC Biotech (GATC, Konstanz, Germany), and the nucleotide sequences were assessed with online similarity searches performed with the Basic Local Alignment Search Tool (BLAST). Isolates were selected for further testing based on the presence of confirmed positive results or an interest in the bacterial species.

### Antimicrobial Susceptibility Testing

M.I.C.Evaluator (Oxoid), ETEST^®^ (bioMérieux, Marcy-l’Étoile, France), or MIC (Liofilchem, Roseto degli Abruzzi, Italy) gradient strips were used to evaluate susceptibility. The following panel of 18 antibiotics spanning 13 classes were used: ampicillin and amoxicillin/clavulanic acid (penicillins); cefotaxime and cefepime (cephalosporins); ciprofloxacin (fluoroquinolones); amikacin, gentamicin, and streptomycin (aminoglycosides); trimethoprim (trimethoprims); trimethoprim/sulfamethoxazole (trimethoprim/sulfonamides); bacitracin (bacitracins); erythromycin (macrolides); tetracycline (tetracyclines); nitrofurantoin (nitrofurans); fosfomycin (fosfomycins); and imipenem and meropenem (carbapenems). Susceptibility profiles to colistin (polymyxins) was determined through the broth micro-dilution method using ComASP^TM^ Colistin (Liofilchem). All testing was performed in accordance to the manufacturer’s instructions. MIC clinical breakpoints from the European Committee on Antimicrobial Susceptibility Testing (EUCAST) were used to determine susceptibility (European Committee on Antimicrobial Susceptibility Testing, 2019). Multidrug resistance was defined as phenotypic resistance to three or more classes of antibiotics.

### Oxford Nanopore MinION Sequencing

DNA library was prepared using the SQK-RBK004 Rapid Barcoding Kit and loaded into the MinION SpotON R9.4.1 flow cell in a MinION MK1 sequencer. The 48 h (MinKNOW v.19.06.8 and older versions) or 72 h (MinKNOW 19.10.1) live basecalling sequencing protocol was selected in the MinKNOW software and allowed to complete basecalling after the completion of the sequencing run. With MinKNOW v.19.06.8 and older, EPI2ME was used to demultiplex the barcodes. With MinKNOW v.19.10.1 and after, barcode demultiplexing was conducted during the run while basecalling. Individual barcodes were examined through the WIMP ARMA applications to identify resistance genes and confirm the species of each isolate. All runs were according to the standard protocol of Oxford Nanopore Technologies (Oxford Nanopore Technologies, Oxford, United Kingdom).

### Illumina MiSeq Sequencing and Assembly

Paired-end libraries (2 × 300 bp) were prepared with NextRra^TM^ DNA Flex Tagmentation (Illumina Inc., San Diego, CA, United States) and sequenced on an Illumina MiSeq platform using a 2 × 300 paired-end approach with v3 chemistry by the Norwegian Sequencing Center (Oslo, Norway).

### Sequence Analysis

The online Galaxy platform was utilized to remove adapters from the MiSeq and MinION Multi FASTQ files with Trimmomatic and Porechop, respectively. MinION data was filtered by sequence length with a minimum threshold set at 800 bp and above. MiSeq and MinION data were combined in Unicycler to assemble sequences into contigs. Additionally, the sequence data was annotated using the NCBI Prokaryotic Genome Annotation Pipeline (Angiuoli et al., 2008). To assess the genomes for acquired antibiotic resistance genes and VFs, ResFinder v 3.2 and VirulenceFinder v 2.0 (Center for Genomic Epidemiology, Technical University of Denmark, Lyngby, Denmark) servers were used with the following settings: selected ID threshold 95%, selected minimum length 60%. The ABRicate Mass screening of contigs for antimicrobial and virulence genes (Galaxy Version 0.9.8) tool combined with the NCBI National Database of Antibiotic Resistant Organisms were also used. Additionally, the Comprehensive Antibiotic Resistance Database (CARD) was used to search the genome for acquired antibiotic resistance genes. MLST v 2.0.4, PlasmidFinder v 2.1, and SerotypeFinder 1.1 (Center for Genomic Epidemiology) were used with default settings to determine MLST type, plasmid types, and serotypes of the isolates, with the exception of strain NMBU_R2 in which the online database PubMLST^1^ (Jolley et al., 2018) was used for MLST results. Phylogenetic groups were determined using the publicly available ClermonTyper^2^. In addition, a custom made virulence database of over 760 genes was employed to assess for VFs with the CGE website. Analysis of WGS results revealed that NMBU_W06E18 was two separate strains rather than a single strain. In this case, MetaSPAdes (Nurk et al., 2017), MaxBin2 (Wu et al., 2016), Unicycler (all from the Galaxy platform), and NCBI Blast were used to separate the strains.

### Water Quality Testing

The water quality of the “Andedammen” site was tested on October 21, 2019 using the Colilert test. It showed a most probable number (MPN) of >24196.6 coliforms and 236 *E. coli* cells per 100 mL of water.

### Nucleotide Sequence Accession Numbers

The sequence data of NMBU_W05E18, NMBU_W07E18, NMBU_W12E19, and NMBU_W10C18 have been deposited in the GenBank database under accession numbers CP042878-CP042881, CP042882-CP042884, CP042885-CP042892, and CP044402-CP044406. NMBU_W06E18 and NMBU_W13E19 have been deposited in the GenBank database under accession numbers CP047609-CP047613, and CP043406-CP043413. NMBU_R2, and NMBU_R16 were deposited at DDBJ/ENA/GenBank under the accession numbers JAAAYF000000000 and JAAAYE000000000, respectively. The versions described in this paper are version JAAAYF01000000 and JAAAYE010000000.

## Results

### Isolation and Characterization of Bacteria

Among the 98 environmental isolates that survived growth on Brilliance^TM^ ESBL or Brilliance^TM^ CRE agar, β-lactamases or ESBLs were detected in eight (8%) isolates. Species identification revealed six isolates within Enterobacteriaceae including five *Escherichia coli* (*E. coli*) strains and one *Klebsiella pneumoniae* (*K. pneumoniae*) strain. Two additional strains were identified within Xanthomonadaceae (*Stenotrophomonas maltophilia*) and Burkholderiaceae (*Paraburkholderia* spp.).

The phylogenetic analysis of the five *E. coli* isolates revealed that the commensal-associated group A and the pathogenic-associated group D were represented. One strain was classified as phylogroup A and ST1286 (Table 2). Four strains were classified as phylogroup D and were of three different ST types: ST405 (*n* = 2), ST69 and ST38. The *K. pneumoniae* isolate was classified as ST659 and *Stenotrophomonas maltophilia* (*S. maltophilia*) as ST31. The ST of the *Paraburkholderia* spp. could not be determined.

**TABLE 2:** Genotypic characteristics of β-lactamase and ESBL-producing isolates, isolation date, site, species, serotype and ST.

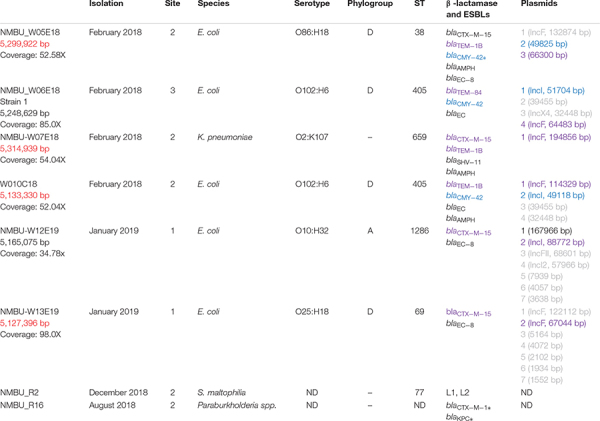

The color of the β-lactamase or ESBL gene coordinates with the color of the plasmid that it is located on, and if the β-lactamase or ESBL is in black, it is chromosomally located. Novel β-lactamases are noted with a *. All strains with a genome size in red are whole genome sequences. Strains with an “E” in the title were isolated from an extended spectrum β-lactam plate and strains with a “C” in the title were isolated from a carbapenem plate.

### Antibiotic Susceptibility Testing

The Enterobacteriaceae isolates showed a range of susceptibility profiles toward 18 antibiotics (Supplementary Table S1). All strains (*n* = 6) were determined to be phenotypically resistant against ampicillin, cefotaxime, cefepime, bacitracin and erythromycin. Resistance to amoxicillin with clavulanic acid (3, 50%), ciprofloxacin (3, 50%), tetracycline (2, 33%), trimethoprim (2, 33%), fosfomycin (1, 16%), and nitrofurantoin (1, 16%) was also observed. No Enterobacteriaceae strains exhibited phenotypic resistance to amikacin, gentamicin, streptomycin, imipenem, meropenem, colistin, or trimethoprim/sulfamethoxazole. It is notable that all of the ESBL-producing Enterobacteriaceae strains were sensitive to carbapenems and colistin, as these are the last resort antibiotics for ESBL-producing bacterial infections in humans. Nevertheless, all Enterobacteriaceae isolates were classified as multidrug-resistant. It is important to note that the WGS results of NMBU_W06E18 indicated that this was a mixed sample of two strains of *E. coli* during susceptibility testing.

Although *S. maltophilia* had high MIC values against many of the tested antibiotics, results for agents other than trimethoprim-sulfamethoxazole should be treated with caution, as EUCAST has not determined clinical breakpoint values for this particular type of bacteria. Therefore, the relationship between various susceptibility testing results and the clinical outcome for *S. maltophilia* infection cannot be confidently determined (Gülmez et al., 2010). However, the MIC values for a variety of antibiotics (streptomycin, imipenem, meropenem, cefotaxime, cefepime, ampicillin, amoxicillin with clavulanic acid, nitrofurantoin, and trimethoprim) exceeded the testable limits, and treatment levels would likely not approach those MIC values. Therefore, this strain was characterized as multidrug resistant. In addition, the *Paraburkholderia* spp. had difficulties growing, thus phenotypic antibiotic susceptibility could not be determined, and additional results that correspond to this strain are limited.

### Detection of β-Lactamases, ESBLs, and Co-resistance Genes

#### Enterobacteriaceae

Data on β-lactamases, ESBLs, and co-resistance genes for all isolates is presented in Tables 2, 3. All Enterobacteriaceae harbored at least one β-lactamase or ESBL gene, with *bla*_CTX–M–15_ (4, 66%) as the most prevalent variant followed by *bla*_TEM–1B_ (3, 50%). Other detected types were *bla*_CMY–42_ (2, 33%) and *bla*_TEM–84_ (1, 16%). Furthermore, a chromosomally located *bla*_CTX–M–15_ was identified in strain NMBU_W05E18, which is a rather new development (Hiari et al., 2013; Rodríguez et al., 2014). All of the *E. coli* strains harbored a genetic region containing an AmpC/CMY-like gene, *blc* and *sugE* as previously described (Verdet et al., 2009; Singh et al., 2018). In strain NMBU-W05E18 this region is situated both on the chromosome and on a plasmid where the NCBI annotation pipeline reports a frameshifted CMY gene, however, on closer inspection a full ORF can be reconstructed by selecting an upstream initiator methionine. This translated ORF shows 99% amino acid identity with CMY-42 with two AA substitutions at codon 303 (Ser303Ile) and at codon 318 (Thr318Ala). No identical proteins were found in BLAST searches; therefore, this may constitute a novel plasmid-borne CMY-variant (Supplementary File S1).

**TABLE 3:** Co-resistance genes identified throughout the isolates.

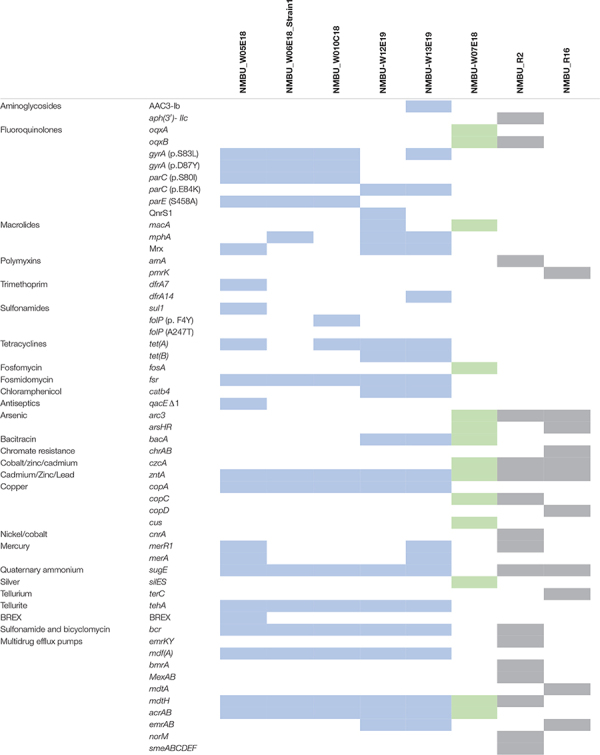

*Present resistance determinants are represented by a gray box. 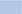 lblue = *E. coli*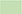 green = *K. pneumoniae*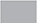 jkgray = *S. maltophilia* and *B. multivorans.**

A wide variety of resistance mechanisms to non-β-lactam drugs were identified amongst the Enterobacteriaceae isolates, including determinants against aminoglycosides [AAC(3)-I]; bacitracin (*bac*); bicyclomycin (*bcr*); chloramphenicol (*catb4*); fluoroquinolones (*oqxA, oqxB, qnrS1*); fosfomycin (*fosA*); fosmidomycin (*fsr*); macrolides (*macA, mphA*, Mrx); sulfonamides (*sul1*); tetracyclines [*tet*(*A*), *tet*(*B*)]; and trimethoprims (*dfrA*). Efflux pumps that confer resistance to multiple classes of antibiotics were also observed (*acrAB, emrAB, mdtH, mdfA*). In addition, several *E. coli* isolates contained chromosomal mutations in *gyrA* (S83L or D87Y), *parC* (S80I, E84K), and *parE* (S458A) that confer fluoroquinolone resistance (Moon et al., 2010; Huseby et al., 2017). Mutations in *folP* (F4Y, A247T) that confer resistance to sulfonamides were also observed (Buwembo et al., 2013). It is notable that no acquired genes encoding carbapenemases or resistance to colistin were found.

Resistance genes against metals were identified, including arsenic (ArsHR), cadmium/zinc/lead (*zntA*), copper (*cus, copA*), mercury (*merA*), and tellurite (*tehA*). In addition, quaternary ammonium is used as a disinfectant in medical and food environments, and the gene that confers that resistance (*sugE*) was observed throughout the isolates (Sundheim et al., 1998). The *qacEΔ1* resistance gene against biocides was also detected in one isolate. Silver resistance determinants (*silES*) were also observed in the *K. pneumoniae* isolate, which is an emerging public health issue, as silver is commonly used as a disinfectant and preservative in a variety of healthcare and consumer products, for instance silver-treated catheters and wound dressings (Silver et al., 2006). Three of the strains were positive for the CRISPR-Cas bacteriophage adaptive immunity system (NMBU_W05E18, NMBU_W13E19 and NMBU_W10C18) and one strain (NMBU_W05E18) additionally contained the novel bacteriophage exclusion (BREX) resistance system, which has an important role in innate defense against phages (Goldfarb et al., 2015).

#### S. maltophilia

Two inducible β-lactamases were found, a class A β-lactamase (L1) and a cephalosporinase (L2). Resistance genes toward aminoglycosides [Aph(3′)- IIc], bicyclomycin (*bcr*), fluoroquinolones (*oqxB*), and polymyxins (*arnA*) were additionally detected. Resistance genes to metals including arsenic (*acr3*), copper (*cop*), cobalt/zinc/cadmium (*czcA*), mercury (*merR1*), nickel/cobalt (*cnrA*), and quaternary ammonium (*sugE*) were observed. In addition, efflux pumps associated with resistance to aminoglycosides, β-lactams, quinolones (SmeABC), chloramphenicol and tetracyclines (SmeDEF) were detected. The multidrug efflux pumps *emrKY*, *mdtH*, *mexAB*, *norM* and *bmrA* were also identified. A resistance mechanism against a plant-produced organic hydroperoxide (*ohr*) was located, which allows for an advantage in the interaction of bacterial pathogens over symbionts in plant hosts (Caswell et al., 2012). The persistence and stress resistance toxin, *pasT*, was identified and is documented to have a role in persistence after antibiotic exposure and survival of nitrosative stress.

#### *Paraburkholderia* spp.

This isolate harbored novel *bla*_CTX–M_-like and *bla*_KPC_-like genes. As there were no identical proteins found on BLAST searches, these may constitute novel plasmid-borne variants (Supplementary File S1). In addition, this strain contained resistance genes toward polymyxins (*pmrK*), arsenic (*arsHR*, *acr3*), chromate resistance (*chrAB*), cobalt/zinc/cadmium (*czcAD*), copper (*copCD*), tellurium resistance (*terC*), and quaternary ammonium (*sugE*). The multidrug efflux pumps *ermAB* and *mdtABC* were also identified. In addition, the “Bacterial abortive infection” system was observed, which activates cell death upon phage infection thereby limiting viral replication and protecting the bacterial population (Dy et al., 2014).

### Detection of Virulence Factors

#### Enterobacteriaceae

All isolates showed a wide array of UPEC-associated virulence genes, including iron acquisition systems (*chuA, fyuA, iroN, irp1, irp2, iuc, iutA, sitA*), protectins (*kpsS, kpsM*), serum resistance (*iss, traT*), adhesins (*csg, eilA, dr/afa, fdeC, fimH, iha, pap*), and toxins (*astA, hlyD, sat, vat, hbp*) (Supplementary Table S2).

In a previous study, we found that fecal samples from Norwegian patients with signs of intestinal infection were colonized with a surprisingly high frequency of intestinal pathogenic *E. coli* (IPEC)/extraintestinal pathogenic *E. coli* (ExPEC) heteropathogenic strains (Lindstedt et al., 2018). Now, we have isolated multidrug resistant heteropathogenics from environmental samples. The *E. coli* surface water strain NMBU_W05E18 [O86:H18 (ST38 – phylogroup D)] contains the *afa* gene cluster that encodes afimbrial adhesins, expressed by both uropathogenic and diarrhea-associated *E. coli* strains and is also associated with mortality due to in bacteraemia infections (Daga et al., 2019). Interestingly, this strain has an *afa* gene cluster both chromosomally and plasmid located. The genomes of *E. coli* strains NMBU_W05E18 and NMBU_W12E19 [O10:H32 (ST1286 – phylogroup A)] contained the *pap* (pyelonephritis-associated pili) operon associated with UPEC strains. All *E. coli* strains except NMBU_W12E19 harbored *eilA* and all *E. coli* strains except NMBU_W10C18 [O102:H6 (ST405 – phylogroup D)] harbored *ygeH*, which are both hilA-like regulator genes, with the HilA protein as the master regulator of the *Salmonella* pathogenicity island 1. The *eilA* gene appears to be restricted to *E. coli* 042 and other enteroaggregative strains, while *ygeH* is present in a variety of *E. coli* strains including K12, enterohemorrhagic O157:H7, enteroaggregative hemorrhagic O104:H4, among others (Hüttener et al., 2014).

In all phylogroup D strains of this study, the *eilA* gene is located in close proximity with genes encoding Air (an enteroaggregative immunoglobulin repeat protein), an IpaD/SipD/SspD family (type III secretion system needle tip protein), and SipB (type III cell invasion protein). However, the translated Air protein is different between the strains, where the protein in NMBU_W06E18_Strain1 and NMBU_W10C18 has a length of 3806 AA and a length of 3418 AA and 4485 AA in NMBU_W13E19 and NMBU_W05E18, respectively. The difference in protein length seems to be attributed to different number of bacterial Ig-like domain 1 (BIG-1 domain) units in the Air proteins. The genomes of the phylogroup D strains also contain genes identified as effector proteins of a type III secretion system (T3SS) in enterohemorrhagic *E. coli* (EHEC) and enteropathogenic *E. coli* (EPEC): *espX1, espX4, espX5, espY1, espY3*, and *espY4*. The EspY1 and EspY4 proteins have been confirmed as genuine translocated T3SS effectors of EHEC. EspY1 was shown to participate in apoptosis and regulation of cell cycle. It has further been demonstrated that EspY3 is an effector protein translocated by the T3SS of both EHEC O157:H7 and EPEC O127:H6, and that EspY3 localizes in the pedestal region in EPEC (Larzábal et al., 2018). The phylogroup A strain (NMBU_ W12E19) harbored *espX1, espX4 and espX5* but *espY1, espY3* and *espY4* were absent. The T3SS effector leucine-rich repeat protein EspR1 was present in strains NMBU_ W12E19 and NMBU_ W13E19, while only strain NMBU_W13E19 was positive for the EspR2 protein. Strain NMBU_W13E19 displayed the gene combination *eilA*, Air and *lpfA* (encoding for long polar fimbriae) that was recently reported from a *bla*_CTX–M_-producing *E. coli* strain isolated from a wastewater treatment plant in China (Jiang et al., 2019) and in eight food-producing animal isolates (seven turkey and one laying hen) in Poland (Zajac et al., 2019). This particular gene combination was also seen in the first clinical isolate of an *E. coli* harboring the *mcr*-*1* gene in Mexico, isolated in 2017 (Merida-Vieyra et al., 2019), as well as in six ESBL-producing *E. coli* strains isolated from veterinary hospital staff and students in three United Kingdom veterinary hospitals (Royden et al., 2019). The NMBU_W12E19 strain has an unusual array of virulence genes when compared to typical and atypical EAEC strains. It has the dispersin locus associated with typical EAEC but is also negative for AggR, which is often used to define a typical EAEC strain. NMBU_W12E19 further harbors the enteroaggregative *E. coli* heat-stable enterotoxin 1 (EAST1), which was originally discovered in EAEC but has also been associated with enterotoxigenic *E. coli* (ETEC). The NMBU_W12E19 *astA* gene has 100% nucleotide identity to the previously described *astA*-allele-2 that was proven to express enterotoxic activity (access nr. AF143819) and has the same genetic location on an insertion sequence (IS) element lying entirely within a transposase-like gene (McVeigh et al., 2000). Interestingly, NMBU_W12E19 is also positive for the *bfpA* and *bfpB* genes of the bundle forming pilus (BFP) associated with typical EPEC (tEPEC), which are often used as genetic markers in intestinal pathogenic *E. coli* clinical diagnostic procedures. The genomic sequences of two O157:H7 strains, EDL933 and Sakai, contain a gene cluster predicted to encode an additional T3SS named ETT2, which is involved in virulence (Zhou et al., 2014). Strain NMBU_W05E18 and NMBU_ W13E19 carry an almost intact ETT2 where only the *eivH* gene is missing. Strain NMBU_ W12E19 carries a deleted ETT2 version where the genes between *epaO* and ECs3736/*pkgA* are deleted, *eivH* is, however, present. In strains NMBU_W10C18 and NMBU_W06E18_Strain1 almost the entire ETT2 locus is deleted between ECs3737 and *yqeH*.

*K. pneumonia* harbored several VFs including those that aid aerobactin transport (*entB, iutA*) as well as the enterobactin/salmochelin importer (*fepB*) which is required to establish an infection in iron poor areas of the body, as in the urinary tract or lungs (Palacios et al., 2017). Major adhesive structures type 1 fimbriae (*fimA*) were present and are expressed in the bladder and have been shown to contribute to uropathogenicity (Paczosa and Mecsas, 2016). Type 3 (*mrk*) fimbrial adhesins were also detected and play an important role in adhesion to medical devices, such as in catheters (Stahlhut et al., 2012). An AcrAB efflux pump was also identified and is recognized as a required virulence factor to resist immune defense mechanisms of the lung, thus facilitating the onset of pneumonia (Padilla et al., 2010). In addition, *kvgAS* was detected which has only been found in virulent *K. pneumoniae* CG43 (Lai et al., 2003).

#### S. maltophilia

This isolate harbored an aerobactin receptor (*fep*), iron receptor (*iroN*), and a gene that aids in the release of iron from other siderophores (*viuB*), which are all imperative to establishing extraintestinal infections. The type II secretion system (*gsp*) was detected that likely plays an important role in pathogenesis of the lungs (Karaba et al., 2013.). In addition, a twitching motility protein (*pilG*) was identified that is known to promote attachment and translocation across host cells (Mattick et al., 1996).

#### Paraburkholderia spp.

No known VFs were detected in this isolate.

#### Plasmid Identification

The IncFII plasmid type was observed to be the most prevalent (*n* = 5). Four plasmids of the IncF family were identified: IncFIA, IncFIB, IncFII, and all strains characterized as phylogroup D carried either or both IncFIB or IncFII. No plasmids could be identified in the *Paraburkholderia* spp. or *S. maltophilia* strains.

## Discussion

Our study aimed to explore the presence of ESBL-producing bacteria in freshwater of Norway, a country with a low prevalence of antibiotic usage and resistance (NORM/NORM-VET, 2018). In addition, we wished to examine the presence of the most clinically-relevant β-lactamases and carbapenemases. An array of ESBL-producing microorganisms were found, with Enterobacteriaceae as the most predominant, and included strains of *E. coli* and *K. pneumoniae*. ESBL-producing Enterobacteriaceae are identified as a serious public health risk and recognized by WHO as pathogens of critical priority amongst MDR bacteria (World Health Organization [WHO], 2014) with community-acquired UTIs as the most common infection (Pitout and Laupland, 2008). In addition to Enterobacteriaceae, β-lactamase and ESBL-producing *S. maltophilia* and a *Paraburkholderia* spp. were observed. *S. maltophilia* has largely been distinguished as a nosocomial pathogen that mainly affects immunocompromised individuals (Spencer, 1995), however, community-acquired infections have been reported. For instance, it has been implicated in lower respiratory tract infections perceived to be due to recreational activity in aquatic environments (Gajdács and Urbán, 2019). The *Paraburkholderia* lineage is unlikely to cause infection (Eberl and Vandamme, 2016), however, the ESBLs harbored by this strain can transfer to pathogenic counterparts.

It is concerning that all tested isolates in our study exhibited a multidrug resistance profile, including to some of the most commonly prescribed antibiotics in clinical settings worldwide. A very high rate of resistance toward ampicillin (100%), bacitracin (100%), erythromycin (100%), and 3rd and 4th generation cephalosporins (100%) was found. Fortunately, no acquired resistance to the last-resort treatment options of carbapenems or colistin was observed. However, a recent study has confirmed the presence of plasmid-mediated colistin-resistant ESBL-producing *E. coli* in a Norwegian public beach (Jørgensen et al., 2017), indicating a risk of dispersal.

Another alarming finding was that a majority of our isolates possessed a multitude of virulence traits central to pathogenicity, namely those necessary to cause UTIs. This is important, as previous research has established that swimming in freshwater is a risk factor for community acquired UTIs with Enterobacteriaceae (Søraas et al., 2013). Although the locations in this study are not regularly used for recreational purposes, swimming does occur on occasion. In addition, water uptake by wild or companion animals can disseminate these pathogens to other locations that have a greater extent of human contact. Another interesting finding was the heteropathogenic nature of various *E. coli* strains. The emergence of heteropathogens may have the potential to lead to serious consequences to public health due to their enhanced virulence capabilities from different pathotypes. In our study, two isolates were characterized as DAEC/UPEC or aEAEC/UPEC and therefore have the potential to cause both diarrheal disease and UTIs. However, the majority of the *E. coli* isolates were characterized solely as UPEC, which is in agreement with a previous study that determined ExPEC as the main pathotype of *E. coli* isolates from water sources (Hamelin et al., 2006). An interesting finding was that three *E. coli* strains contained the *fec* locus, making them potential mastitis-causing strains in bovines, a disease that has a significant economic impact on global dairy production (Blum et al., 2018). A worrisome finding was that a serious multidrug resistant strain of similar ST as our multidrug resistant *S. maltophilia* of ST31 was detected from a human blood sample in China (Zhao et al., 2015). Additionally, the ST38, ST69, and ST405 types detected in this study were also observed in human clinical samples throughout Norway (Lin T Brandal pers. comm., Enterobase database search), which further connected the clinical relevance of some of our environmental isolates.

The *bla*_CTX–M–15_ enzyme was the predominant variant detected in this study, and is currently the most prevalent ESBL in clinical isolates (Livermore and Hawkey, 2005), human and animal feces (Guenther et al., 2011), and aquatic environments across the world (Zurfluh et al., 2013; Zarfel et al., 2017). As in our study, other research has determined that *bla*_CTX–M–15_ is strongly associated with human ExPEC strains, particularly UPEC (Mellata, 2013). An exciting result was the detection of the novel variants *bla*_CMY–42_-like, *bla*_CTX–M_-_1_-like, and *bla*_KPC_-like which highlights the importance of monitoring the evolution of β-lactamase and ESBL-producing bacteria. In addition, one isolate contained a chromosomally located *bla*_CTX–M–15_ (ST38). Previous studies have identified a strong relationship between ST38 and a chromosomally located *bla*_CTX–M–15_ (Dimou et al., 2012; Rodríguez et al., 2014), not only in clinical settings (Coque et al., 2008), but in Mongolian wild birds as well (Guenther et al., 2017). As there is abundant wildlife around the sample locations, the wild birds may be a potential explanation for the source of the ESBL-producing isolates in this study. The investigated site “Andedammen” means “duck pond” and is a common gathering locale for wild birds that may have influenced the bacterial community with bird droppings. To supplement that speculation, previous studies have determined that ESBL-producing bacteria from both wild birds and humans are genetically similar (Bonnedahl et al., 2014; Atterby et al., 2017). It is worrisome that the resistance determinants are commonly located on IncF-type plasmids, as they represent a serious risk for the further dissemination of resistance genes. The presence of IncF-type plasmids in a rich reservoir of freshwater increases the likelihood of acquiring additional resistance determinants as well as the evolution of novel resistance determinants (Amos et al., 2014).

## Conclusion

The present study confirms the existence of multidrug resistant ESBL-producing bacteria in aquatic environments of Norway, a country with a low incidence of antibiotic resistance. It is also worrisome that the majority of the isolates contained virulence determinants of major pathogens and STs seen in human patient isolates, indicating the potential to cause serious infections. As humans can be exposed through the food chain or recreational activities, the presence of ESBLs in the environment pose a critical threat to global public health, and our results provide additional support to this epidemiological association.

## Data Availability Statement

The datasets generated for this study can be found in the GenBank: CP042878-CP042881, CP042882-CP042884, CP042885-CP042892, CP044402-CP044406, CP047609-CP047613, and CP043406-CP043413. DDBJ/ENA/GenBank: JAAAYF000000000 and JAAAYE000000000.

## Author Contributions

MF carried out the experimental work, performed bioinformatics, analyzed the data, and wrote the original draft of the manuscript. B-AL conceptualized the experimental methods, performed bioinformatics, analyzed the data, and participated in the review and editing of the manuscript. RM and DP performed the bioinformatics and participated in the review and editing of the manuscript. LB participated in the review and editing of the manuscript.

## Conflict of Interest

The authors declare that the research was conducted in the absence of any commercial or financial relationships that could be construed as a potential conflict of interest.

## Footnotes

1 www.pubmlst.org

2 http://clermontyping.iame-research.center/
