# Supplementary material for: Whole Genome Sequencing and Characterization of Multidrug-Resistant (MDR) Bacterial Strains Isolated From a Norwegian University Campus Pond
Source: Front Microbiol. 2020 Jun 17;11:1273. doi: 10.3389/fmicb.2020.01273 (PMC7311804; doi:10.3389/fmicb.2020.01273)

**Supplemental file 1.**

Novel CMY-42 variant gene.

**NMBU_W05E18 CMY-42-variant gene: ORF nucleotide sequence (ACCESSION number CP042878).**

ATGATGAAAAAATCGTTATGCTGCGCTCTGCTGCTGACAGCCTCTTTCTCCACATTTGCTGCCGCAAAAA

CAGAACAACAGATTGCCGATATCGTTAATCGCACCATCACCCCGTTGATGCAGGAGCAGGCTATTCCGGG

TATGGCCGTTGCCGTTATCTACCAGGGAAAACCCTATTATTTCACCTGGGGTAAAGCCGATATCGCCAAT

AACCACCCAGTCACGCAGCAAACGCTGTTTGAGCTAGGATCGGTTAGTAAGACGTTTAACGGCGTGTTGG

GCGGCGATGCTATCGCCCGCGGCGAAATTAAGCTCAGCGATCCGGTCACGAAATACTGGCCAGAACTGAC

AGGCAAACAGTGGCAGGGTATCCGCCTGCTGCACTTAGCCACCTATACGGCAGGCGGCCTACCGCTGCAG

ATCCCCGATGACGTTAGGGATAAAGCCGCATTACTGCATTTTTATCAAAACTGGCAGCCGCAATGGACTC

CGGGCGCTAAGCGACTTTACGCTAACTCCAGCATTGGTCTGTTTGGCGCGCTGGCGGTGAAACCCTCAGG

AATGAGTTACGAAGAGGCAATGACCAGACGCGTCCTGCAACCATTAAAACTGGCGCATACCTGGATTACG

GTTCCGCAGAACGAACAAAAAGATTATGCCTGGGGCTATCGCGAAGGGAAGCCCGTACACAGTTCTCCGG

GACAACTTGACGCCGAAGCCTATGGCGTGAAATCCAGCGTTATTGATATGGCCCGCTGGGTTCAGGCCAA

CATGGATGCCAGCCACGTTCAGGAGAAAACGCTCCAGCAGGGCATTGCGCTTGCGCAGTCTCGCTACTGG

CGTATTGGCGATATGTACCAGGGATTAGGCTGGGAGATGCTGAACTGGCCGCTGAAAGCTGATTCTAGCA

TCAACGGCAGCGACAGCAAAGTGGCATTGGCAGCGCTTCCCACCGTTGAGGTAAACCCGCCCGCCCCCGC

AGTGAAAGCCTCATGGGTGCATAAAACGGGCTCCACTGGTGGATTTGGCAGCTACGTAGCCTTCGTTCCA

GAAAAAAACCTTGGCATCGTGATGCTGGCAAACAAAAGCTATCCTAACCCTGTCCGTGTCGAGGCGGCCT

GGCGCATTCTTGAAAAGCTGCAATAA

**NMBU_W05E18 CMY-42-variant gene: translated amino acid sequence.**

**SEQUENCE 381 AA.**

MMKKSLCCAL LLTASFSTFA AAKTEQQIAD IVNRTITPLM QEQAIPGMAV AVIYQGKPYY

FTWGKADIAN NHPVTQQTLF ELGSVSKTFN GVLGGDAIAR GEIKLSDPVT KYWPELTGKQ

WQGIRLLHLA TYTAGGLPLQ IPDDVRDKAA LLHFYQNWQP QWTPGAKRLY ANSSIGLFGA

LAVKPSGMSY EEAMTRRVLQ PLKLAHTWIT VPQNEQKDYA WGYREGKPVH SSPGQLDAEA

YGVKSSVIDM ARWVQANMDA SHVQEKTLQQ GIALAQSRYW RIGDMYQGLG WEMLNWPLKA

DSSINGSDSK VALAALPTVE VNPPAPAVKA SWVHKTGSTG GFGSYVAFVP EKNLGIVMLA

NKSYPNPVRV EAAWRILEKL Q

**Alignment of beta-lactamases from environmental strains and references (CLUSTAL Omega v.1.2.4).**

CMY-variant_NMBU_W05E18 MMKKSLCCALLLTASFSTFAAAKTEQQIADIVNRTITPLMQEQAIPGMAVAVIYQGKPYY 60

F7O57_00310 MMKKSLCCALLLTASFSTFAAAKTEQQIADIVNRTITPLMQEQAIPGMAVAVIYQGKPYY 60

CMY_NMBU_W06E18 MMKKSLCCALLLTASFSTFAAAKTEQQIADIVNRTITPLMQEQAIPGMAVAVIYQGKPYY 60

AID23886.1 MMKKSLCCALLLTASFSTFAAAKTEQQIADIVNRTITPLMQEQAIPGMAVAVIYQGKPYY 60

AOG62294.1 MMKKSLCCALLLTASFSTFAAAKTEQQIADIVNRTITPLMQEQAIPGMAVAVIYQGKPYY 60

WP_063610930.1 -MFKTTLCALLITASCSTFAA---PQQINDIVHRTITPLIEQQKIPGMAVAVIYQGKPYY 56

FVP48_14090 -MFKTTLCALLITASCSTFAA---PQ**K**INDIVHRTITPLIEQQKIPGMAVAVIYQGKPYY 56

NP_313158.1 -MFKTTLCALLITASCSTFAA---PQQINDIVHRTITPLIEQQKIPGMAVAVIYQGKPYY 56

FVA71_23515 -MFKMTLCALLITASCSTFAA---PQQINDIVHRTITPLIEQQKIPGMAVAVIYQGKPYY 56

FZN32_14220 -MFKMTLCALLITASCSTFAA---PQQINDIVHRTITPLIEQQKIPGMAVAVIYQGKPYY 56

F7O57_02980 -MFKMTLCALLITASCSTFAA---PQQINDIVHRTITPLIEQQKIPGMAVAVIYQGKPYY 56

beta_lactamase_C_NMBU_W06E18 -MFKMTLCALLITASCSTFAA---PQQINDIVHRTITPLIEQQKIPGMAVAVIYQGKPYY 56

WP_063860454.1 -MFKTTLCALLITASCSTFAA---PQQINDIVHRTITPLIEQQKIPGMAVAVIYQGKPYY 56

NP_418574.1 -MFKTTLCALLITASCSTFAA---PQQINDIVHRTITPLIEQQKIPGMAVAVIYQGKPYY 56

* * ****:*** ***** *:* ***:******:::* ****************

CMY-variant_NMBU_W05E18 FTWGKADIANNHPVTQQTLFELGSVSKTFNGVLGGDAIARGEIKLSDPVTKYWPELTGKQ 120

F7O57_00310 FTWGKADIANNHPVTQQTLFELGSVSKTFNGVLGGDAIARGEIKLSDPVTKYWPELTGKQ 120

CMY_NMBU_W06E18 FTWGKADIANNHPVTQQTLFELGSVSKTFNGVLGGDAIARGEIKLSDPVTKYWPELTGKQ 120

AID23886.1 FTWGKADIANNHPVTQQTLFELGSVSKTFNGVLGGDAIARGEIKLSDPVTKYWPELTGKQ 120

AOG62294.1 FTWGKADIANNHPVTQQTLFELGSVSKTFNGVLGGDAIARGEIKLSDPVTKYWPELTGKQ 120

WP_063610930.1 FTWGYADIAKKQPVTQQTLFELGSVSKTFTGVLGGDAIARGEIKLSDPATKYWPELTAKQ 116

FVP48_14090 FTWGYADIAKKQPVTQQTLFELGSVSKTFTGVLGGDAIARGEIKLSDPATKYWPELTAKQ 116

NP_313158.1 FTWGYADIAKKQPVTQQTLFELGSVSKTFTGVLGGDAIARGEIKLSDPATKYWPELTAKQ 116

FVA71_23515 FTWGYADIAKKQPVTQQTLFELGSVSKTFTGVLGGDAIARGEIKLSDPATKYWPELTAKQ 116

FZN32_14220 FTWGYADIAKKQPVTQQTLFELGSVSKTFTGVLGGDAIARGEIKLSDPATKYWPELTAKQ 116

F7O57_02980 FTWGYADIAKKQPVTQQTLFELGSVSKTFTGVLGGDAIARGEIKL**I**DPATKYWPELTAKQ 116

beta_lactamase_C_NMBU_W06E18 FTWGYADIAKKQPVTQQTLFELGSVSKTFTGVLGGDAIARGEIKL**I**DPATKYWPELTAKQ 116

WP_063860454.1 FTWGYADIAKKQPVTQQTLFELGSVSKTFTGVLGGDAIARGEIKLSDPATKYWPELTAKQ 116

NP_418574.1 FTWGYADIAKKQPVTQQTLFELGSVSKTFTGVLGGDAIARGEIKLSDP**T**TKYWPELTAKQ 116

**** ****:::*****************.*************** **.********.**

CMY-variant_NMBU_W05E18 WQGIRLLHLATYTAGGLPLQIPDDVRDKAALLHFYQNWQPQWTPGAKRLYANSSIGLFGA 180

F7O57_00310 WQGIRLLHLATYTAGGLPLQIPDDVRDKAALLHFYQNWQPQWTPGAKRLYANSSIGLFGA 180

CMY_NMBU_W06E18 WQGIRLLHLATYTAGGLPLQIPDDVRDKAALLHFYQNWQPQWTPGAKRLYANSSIGLFGA 180

AID23886.1 WQGIRLLHLATYTAGGLPLQIPDDVRDKAALLHFYQNWQPQWTPGAKRLYANSSIGLFGA 180

AOG62294.1 WQGIRLLHLATYTAGGLPLQIPDDVRDKAALLHFYQNWQPQWTPGAKRLYANSSIGLFGA 180

WP_063610930.1 WNGITLLHLATYTAGGLPLQVPD**E**VKSSSDLLRFYQNWQPAWAPGTQRLYANSSIGLFGA 176

FVP48_14090 WNGITLLHLATYTAGGLPLQVPD**E**VKSSSDLLRFYQNWQPAWAPGTQRLYANSSIGLFGA 176

NP_313158.1 WNGITLLHLATYTAGGLPLQVPD**E**VKSSSDLLRFYQNWQPAWAPGTQRLYANSSIGLFGA 176

FVA71_23515 WNGITLLHLATYTAGGLPLQVPD**E**VKSSSDLLRFYQNWQPAWAPGTQRLYANSSIGLFGA 176

FZN32_14220 WNGITLLHLATYTAGGLPLQVPDDVKSSSDLLRFYQNWQPAWAPGTQRLYANSSIGLFGA 176

F7O57_02980 WNGITLLHLATYTAGGLPLQVPDDVKSSSDLLRFYQNWQPAWAPGTQRLYANSSIGLFGA 176

beta_lactamase_C_NMBU_W06E18 WNGITLLHLATYTAGGLPLQVPDDVKSSSDLLRFYQNWQPAWAPGTQRLYANSSIGLFGA 176

WP_063860454.1 WNGITLLHLATYTAGGLPLQVPDDVKSSSDLLRFYQNWQPAWAPGTQRLYANSSIGLFGA 176

NP_418574.1 WNGITLLHLATYTAGGLPLQVPD**E**VKSSSDLLRFYQNWQPAWAPGTQRLYANSSIGLFGA 176

*:** ***************:**:*:..: **:******* *:**::*************

CMY-variant_NMBU_W05E18 LAVKPSGMSYEEAMTRRVLQPLKLAHTWITVPQNEQKDYAWGYREGKPVH**S**SPGQLDAEA 240

F7O57_00310 LAVKPSGMSYEEAMTRRVLQPLKLAHTWITVPQNEQKDYAWGYREGKPVH**S**SPGQLDAEA 240

CMY_NMBU_W06E18 LAVKPSGMSYEEAMTRRVLQPLKLAHTWITVPQNEQKDYAWGYREGKPVH**S**SPGQLDAEA 240

AID23886.1 LAVKPSGMSYEEAMTRRVLQPLKLAHTWITVPQNEQKDYAWGYREGKPVH**S**SPGQLDAEA 240

AOG62294.1 LAVKPSGMSYEEAMTRRVLQPLKLAHTWITVPQNEQKDYAWGYREGKPVHVSPGQLDAEA 240

WP_063610930.1 LAVKPSGLSFEQAMQTRVFQPLKLNHTWINVPP**P**EEKNYAWGYREGKAVHVSPGALDAEA 236

FVP48_14090 LAVKPSGLSFEQAMQTRVFQPLKLNHTWINVPPAEEKNYAWGYREGKAVHVSPGALDAE**T** 236

NP_313158.1 LAVKPSGLSFEQAMQTRVFQPLKLNHTWINVPPAEEKNYAWGYREGKAVHVSPGALDAE**T** 236

FVA71_23515 LAVKPSGLSFEQAMQTRVFQPLKL**T**HTWINVP**S**AEEKNYAWGYREGKAVHVSPGALDAEA 236

FZN32_14220 LAVKPSGLSFEQAMQTRVFQPLKL**T**HTWINVP**S**AEEKNYAWGYREGKAVHVSPGALDAEA 236

F7O57_02980 LAVKPSGLSFEQAMQTRVF**H**PLKL**T**HTWINVP**S**AEEKNYAWGYREGKAVHVSPGALDAEA 236

beta_lactamase_C_NMBU_W06E18 LAVKPSGLSFEQAMQTRVF**H**PLKL**T**HTWINVP**S**AEEKNYAWGYREGKAVHVSPGALDAEA 236

WP_063860454.1 LAVKPSGLSFEQAMQTRVFQPLKLNHTWINVPPAEEKNYAWGYREGKAVHVSPGALDAEA 236

NP_418574.1 LAVKPSGLSFEQAMQTRVFQPLKLNHTWINVPPAEEKNYAWGYREGKAVHVSPGALDAEA 236

*******:*:*:** **::**** ****.** *:*:********* ** *** ****:

CMY-variant_NMBU_W05E18 YGVKSSVIDMARWVQANMDASHVQEKTLQQGIALAQSRYWRIGDMYQGLGWEMLNWPLKA 300

F7O57_00310 YGVKSSVIDMARWVQANMDASHVQEKTLQQGIALAQSRYWRIGDMYQGLGWEMLNWPLKA 300

CMY_NMBU_W06E18 YGVKSSVIDMARWVQANMDASHVQEKTLQQGIALAQSRYWRIGDMYQGLGWEMLNWPLKA 300

AID23886.1 YGVKSSVIDMARWVQANMDASHVQEKTLQQGIALAQSRYWRIGDMYQGLGWEMLNWPLKA 300

AOG62294.1 YGVKSSVIDMARWVQANMDASHVQEKTLQQGIALAQSRYWRIGDMYQGLGWEMLNWPLKA 300

WP_063610930.1 YGVKSTIEDMARWV**R**SNM**N**P**R**DI**ND**KTLQQGIQLAQSRYWQTGDMYQGLGWEMLDWPVNP 296

FVP48_14090 YGVKSTIEDMA**C**WV**R**SNM**N**P**R**DI**ND**KTLQQGIQLAQSRYWQTGDMYQGLGWEMLDWPVNP 296

NP_313158.1 YGVKSTIEDMA**C**WV**R**SNM**N**P**R**DI**ND**KTLQQGIQLAQSRYWQTGDMYQGLGWEMLDWPVNP 296

FVA71_23515 YGVKSTIEDMARWVQSN**L**KPLDITEKTLQQGIQLAQSRYWQTGDMYQGLGWEMLDWPVNP 296

FZN32_14220 YGVKSTIEDMARWVQSN**L**KPLDITEKTLQQGIQLAQSRYWQTGDMYQGLGWEMLDWPVNP 296

F7O57_02980 YGVKSTIEDMARWVQSN**L**KPLDITEKTLQQGIQLAQSRYWQTGDMYQGLGWEMLDWPVNP 296

beta_lactamase_C_NMBU_W06E18 YGVKSTIEDMARWVQSN**L**KPLDITEKTLQQGIQLAQSRYWQTGDMYQGLGWEMLDWPVNP 296

WP_063860454.1 YGVKSTIEDMARWVQSN**L**KPLDITEKTLQQGIQLAQSRYWQTGDMYQGLGWEMLDWPVNP 296

NP_418574.1 YGVKSTIEDMARWVQSN**L**KPLDI**N**EKTLQQGIQLAQSRYWQTGDMYQGLGWEMLDWPVNP 296

*****:: *** **::*:. .: :******* *******: ************:**::

CMY-variant_NMBU_W05E18 DS**S**INGSDSKVALAALP**T**VEVNPPAPAVKASWVHKTGSTGGFGSYVAFVPEKNLGIVMLA 360

F7O57_00310 DSIINGSDSKVALAALPAVEVNPPAPAVKASWVHKTGSTGGFGSYVAFVPEKNLGIVMLA 360

CMY_NMBU_W06E18 DSIINGSDSKVALAALPAVEVNPPAPAVKASWVHKTGSTGGFGSYVAFVPEKNLGIVMLA 360

AID23886.1 DSIINGSDSKVALAALPAVEVNPPAPAVKASWVHKTGSTGGFGSYVAFVPEKNLGIVMLA 360

AOG62294.1 DSIINGSDSKVALAALPAVEVNPPAPAVKASWVHKTGSTGGFGSYVAFVPEKNLGIVMLA 360

WP_063610930.1 DSIINGS**G**NKIALAA**P**PVKAITPPTPAVRASWVHKTGATGGFGSYVAFIPEKELGIVMLA 356

FVP48_14090 DSIINGS**G**NKIALAARPVKAITPPTPAVRASWVHKTGATGGFGSYVAFIPEKELGIVMLA 356

NP_313158.1 D**I**IVNGSDNKIALAARPVKAITPPTPAVRASWVHKTGATGGFGSYVAFIPEKELGIVMLA 356

FVA71_23515 D**I**IINGSDNKIALAARPVKAITPP**A**PAVRASWVHKTGATGGFGSYVAFIPEKELGIVMLA 356

FZN32_14220 D**I**IINGSDNKIALAARPVKAITPPTPAV**C**ASWVHKTGATGGFGSYVAFIPEKELGIVMLA 356

F7O57_02980 D**I**IINGSDNKIALAARPVKAITPPTPAVRASWVHKTGATGGFGSYVAFIPEKELGIVMLA 356

beta_lactamase_C_NMBU_W06E18 D**I**IINGSDNKIALAARPVKAITPPTPAVRASWVHKTGATGGFGSYVAFIPEKELGIVMLA 356

WP_063860454.1 DSIINGSDNKIALAARPVKAITPPTPAVRASWVHKTGATGGFGSYVAFIPEKELGIVMLA 356

NP_418574.1 DSIINGSDNKIALAARPVKAITPPTPAVRASWVHKTGATGGFGSYVAFIPEKELGIVMLA 356

* :***..*:**** *. :.**:*** ********:**********:***:*******

CMY-variant_NMBU_W05E18 NKSYPNPVRVEAAWRILEKLQ 381

F7O57_00310 NKSYPNPVRVEAAWRILEKLQ 381

CMY_NMBU_W06E18 NKSYPNPVRVEAAWRILEKLQ 381

AID23886.1 NKSYPNPVRVEAAWRILEKLQ 381

AOG62294.1 NKSYPNPVRVEAAWRILEKLQ 381

WP_063610930.1 NKNYPNPARVAAAWQILNALQ 377

FVP48_14090 NKNYPNPARV**T**AAWQILNALQ 377

NP_313158.1 NKNYPNPARVAAAWQILNALQ 377

FVA71_23515 NKNYPNPARVAAAWQILNALQ 377

FZN32_14220 NKNYPNPARV**T**AAWQILNALQ 377

F7O57_02980 NKNYPNPARVAAAWQILNALQ 377

beta_lactamase_C_NMBU_W06E18 NKNYPNPARVAAAWQILNALQ 377

WP_063860454.1 NKNYPNPARV**T**AAWQILNALQ 377

NP_418574.1 NKNYPNPARV**D**AAWQILNALQ 377

Legend:

WP_063860454.1: cephalosporin-hydrolyzing class C beta-lactamase EC-8 [Escherichia coli]

WP_063610930.1: class C extended-spectrum beta-lactamase EC-15 [Escherichia coli]

AID23886.1: beta-lactamase CMY-42 [Escherichia coli]

AOG62294.1: CMY-2 [Escherichia coli]

NP_313158.1: beta-lactamase [Escherichia coli O157:H7 str. Sakai]

NP_418574.1 beta-lactamase [Escherichia coli str. K-12 substr. MG1655]

FVP48_14090: class C beta-lactamase from NMBU-W12E19

FVA71_23515: class C beta-lactamase from NMBU_W05E18 (chromosome)

FZN32_14220: class C beta-lactamase from NMBU-W13E19

F7O57_00310: class C beta-lactamase from NMBU-W10C18 (plasmid)

F7O57_02980: class C beta-lactamase from NMBU-W10C18 (chromosome)

beta_lactamase_C_NMBU_W06E18: class C beta-lactamase from NMBU_W06E18

**CMY-variant_NMBU_W05E18: New CMY-42 variant from NMBU_W05E18 (plasmid)**

**Alignment of two novel class-A β-lactamases from strain NMBU_R16 (bla_1 and bla_2) with closest matches.**

**bla_1)** Query = NMBU_R16_locus_tag="GWC77_08380"

Sbjct = class A beta-lactamase [Burkholderia sp. AD24], Sequence ID: WP_134964008.1


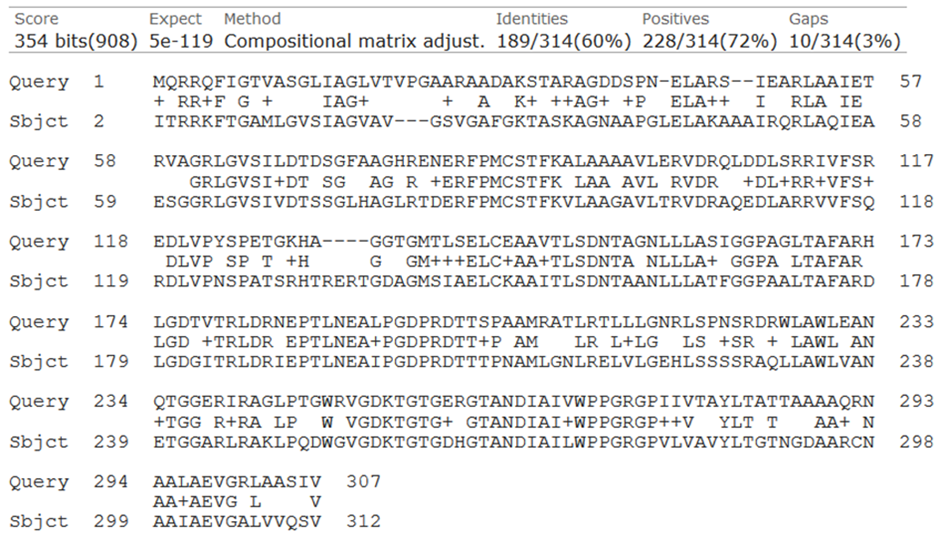


**bla_2**) Query = NMBU_R16_locus_tag="GWC77_24470"

Sbjct = class A beta-lactamase [Paraburkholderia sp. DHOM06], Sequence ID: WP_115533798.1


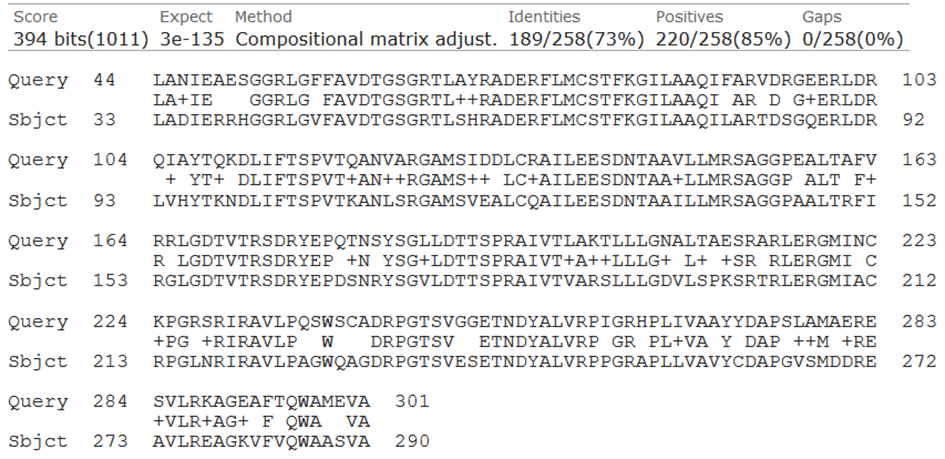

Supplement: Supplementary file 1 [file Data_Sheet_1.docx]
