# Supplementary material for: Whole Genome Sequencing and Characterization of Multidrug-Resistant (MDR) Bacterial Strains Isolated From a Norwegian University Campus Pond
Source: Front Microbiol. 2020 Jun 17;11:1273. doi: 10.3389/fmicb.2020.01273 (PMC7311804; doi:10.3389/fmicb.2020.01273)
Supplement: Supplementary file 3 [file Table_2.docx]

**Supplementary table 2**. Virulence gene profiles of *E. coli* isolates. Gray blocks represent the presence of a virulence gene.

| **VIRULENCE GENE** | **ACCESSION NUMBER** | **DESCRIPTION** | **NMBU_W05E18** | **NMBU_W06E18_Strain 1** | **NMBU_W10C18** | **NMBU_W12E19** | **NMBU_W13E19** |
| --- | --- | --- | --- | --- | --- | --- | --- |
| *aapA* | Z32523 | Dispersin (anti-aggregation protein) gene |  |  |  |  |  |
| *aatA* | FN554767 | Dispersin transporter protein |  |  |  |  |  |
| *aatB* | JX402062 | APEC autotransporter adhesin |  |  |  |  |  |
| *aatD* | HE603111 | Dispersin (anti-aggregation protein) gene |  |  |  |  |  |
| *aec35* | AY857617 | Part of a selC-Associated Genomic Island of APEC strain BEN2908 |  |  |  |  |  |
| *aec36* | AY857617 | Putative MFS superfamily hexuronate transporter |  |  |  |  |  |
| *aec37* | AY857617 | Putative glucosidase-family 31 of glycosyl hydrolases |  |  |  |  |  |
| *aec77* | CYDF01000002 | Similarity to YeeW, *E. coli* RW1374 |  |  |  |  |  |
| *afa/Dr* | AY030355 | DNA sequence specific to the C1845 *afa/Dr* DAEC strain |  |  |  |  |  |
| *afaE-2* | X85782 | *afa* E-2 protein, adhesin of tDAEC |  |  |  |  |  |
| AIDA-I | BA000007 | Potent bacterial adhesin that mediates bacterial attachment to a broad variety of human and other mammalian cells |  |  |  |  |  |
| Air | CP003034 | Putative adhesin – (enteroaggregative immunoglobulin repeat protein) |  |  |  |  |  |
| antigen-43 | U24429 | AIDA-I–type AT protein |  |  |  |  |  |
| antigen-43b | AE014075 | AIDA-I–type AT protein |  |  |  |  |  |
| *aslA* | CU928163 | Contributes to Invasion of Brain Microvascular Endothelial Cells In Vitro and In Vivo |  |  |  |  |  |
| *bfpA* | NZ_AIFX01000009 | Bundle-forming pilus associated with typical EPEC |  |  |  |  |  |
| *bfpB* | CABEEG010000008 | Bundle-forming pilus associated with typical EPEC |  |  |  |  |  |
| *cas1* | BA000007 | CRISPR-associated endonuclease |  |  |  |  |  |
| *cas2* | BA000007 | CRISPR-associated protein |  |  |  |  |  |
| *cas3* | BA000007 | CRISPR-associated protein |  |  |  |  |  |
| *casA* | BA000007 | CRISPR system Cascade subunit |  |  |  |  |  |
| *casE* | BA000007 | CRISPR-associated protein Cas6/Cse3/CasE |  |  |  |  |  |
| *cfaB* | CYCQ01000001 | Colonization factor antigen 1 |  |  |  |  |  |
| *chuA* | LT827011 | Outer membrane hemin receptor |  |  |  |  |  |
| *cirA* | CP030337 | Ferric iron-catecholate outer membrane transporter |  |  |  |  |  |
| *csgA* | CP023388 | Major curlin subunit, from *E. coli* strain 1105 |  |  |  |  |  |
| *csgA* | BA000007 | Major curlin subunit, from *E. coli* strain O157:H7 Sakai |  |  |  |  |  |
| *csgB* | CP027060 | Minor curlin subunit |  |  |  |  |  |
| *csgE* | NC_011750 | Curli production assembly/transport component |  |  |  |  |  |
| *csgF* | NC_011750 | Curli production assembly/transport component |  |  |  |  |  |
| *csgG* | CP003034 | Curli production assembly/transport component |  |  |  |  |  |
| *csgG* | LT903847 | Curli production assembly/transport component |  |  |  |  |  |
| *cvaA* | CU928146 | Colicin-V immunity protein |  |  |  |  |  |
| *eaeH* | CP012635 | Putative attaching and effacing protein |  |  |  |  |  |
| *EAST1/astA* | AF143819 | Heat-stable enterotoxin 1 (allele-2) |  |  |  |  |  |
| *ecpA* | BA000007 | Common pilus major fimbrillin subunit |  |  |  |  |  |
| *ecpB* | NZ_QOON01000045 | Probably fimbrial chaperone |  |  |  |  |  |
| *ecpD* | CP019777 | Common pilus fimbria adhesin |  |  |  |  |  |
| *ECs3737* | NC_002695 | ETT2 gene |  |  |  |  |  |
| *ehaA* | BA000007 | Autotransporter protein of EHEC O157:H7 |  |  |  |  |  |
| *ehaB* | BA000007 | The *E. coli* O157:H7 EhaB autotransporter protein binds to laminin and collagen I and induces a serum IgA response in O157:H7 challenged cattle |  |  |  |  |  |
| *ehaC* | BA000007 | Autotransporter that may play a role in aEPEC infection |  |  |  |  |  |
| *eilA* | CP023364 | transcriptional regulator HilA, locus |  |  |  |  |  |
| *eitA* | DQ381420 | putative iron transport system, periplasmic binding protein |  |  |  |  |  |
| *eitB* | DQ381420 | putative iron transport system, permease component |  |  |  |  |  |
| *eitC* | DQ381420 | putative iron transport system, ATP-binding component |  |  |  |  |  |
| *eivC* | KU684470 | ETT2 gene |  |  |  |  |  |
| *eivF* | NC_002695 | ETT2 gene |  |  |  |  |  |
| *eivG* | NC_002695 | ETT2 gene |  |  |  |  |  |
| *eivH* | DQ077151 | ETT2 gene |  |  |  |  |  |
| *eivI* | KU684470 | ETT2 gene |  |  |  |  |  |
| *eivJ* | KU684470 | ETT2 gene |  |  |  |  |  |
| *entA* | CP027060 | Involved in the biosynthesis of the siderophore enterobactin |  |  |  |  |  |
| *entE* | CP027060 | Involved in the biosynthesis of the siderophore enterobactin |  |  |  |  |  |
| *entH* | CP027060 | Involved in the biosynthesis of the siderophore enterobactin |  |  |  |  |  |
| *epaO* | KU684470 | ETT2 gene |  |  |  |  |  |
| *epaP* | NC_002695 | ETT2 gene |  |  |  |  |  |
| *epaQ* | NC_002695 | ETT2 gene |  |  |  |  |  |
| *epaR* | KU684470 | ETT2 gene |  |  |  |  |  |
| *epaS1* | KU684470 | ETT2 gene |  |  |  |  |  |
| *epaS2* | KU684470 | ETT2 gene |  |  |  |  |  |
| *eprH* | NC_011750 | Putative Type III secretion protein |  |  |  |  |  |
| *eprI* | NC_002695 | Type III secretion protein |  |  |  |  |  |
| *eprJ* | KU684470 | Type III secretion apparatus protein |  |  |  |  |  |
| *eprK* | NC_011750 | Type III secretion |  |  |  |  |  |
| *espR1* | BA000007 | Non-LEE-encoded type III secreted effector |  |  |  |  |  |
| *espR2* | BA000007 | Type III secretion effector |  |  |  |  |  |
| *espX1* | CP003034 | Putative type III secreted effector |  |  |  |  |  |
| *espX4* | NC_017646 | Type III secretion system (T3SS) in EHEC and EPEC |  |  |  |  |  |
| *espX5* | NC_017646 | Type III secretion system (T3SS) in EHEC and EPEC |  |  |  |  |  |
| *espY1* | NC_002695 | Type III secretion system (T3SS) in EHEC and EPEC |  |  |  |  |  |
| *espY3* | NC_002695 | Type III secretion system (T3SS) in EHEC and EPEC |  |  |  |  |  |
| *espY4* | NC_002695 | Type III secretion system (T3SS) in EHEC and EPEC |  |  |  |  |  |
| *etrA* | KU684467 | Type III secretion regulator |  |  |  |  |  |
| *fdeC* | CP019777 | Intimin-like protein that was shown to contribute to kidney colonization in a mouse urinary tract infection model |  |  |  |  |  |
| *fecA* | NC_011751 | Outer membrane ferric-siderophore receptor |  |  |  |  |  |
| *feoB* | CP019777 | Ferrous iron uptake system which is active under anaerobic growth conditions |  |  |  |  |  |
| *fepA* | CP000468 | Outer membrane (OM) protein that binds and transports ferric enterobactin (ferric enterochelin) |  |  |  |  |  |
| *fepB* | AE014075 | Binds ferrienterobactin; part of the binding-protein-dependent transport system for uptake of ferrienterobactin |  |  |  |  |  |
| *fepC* | AE014075 | Encodes the predicted ATP-binding subunit of a ferric enterobactin ABC transporter complex |  |  |  |  |  |
| *fepD* | AE014075 | Subunit of a ferric enterobactin ABC transporter complex |  |  |  |  |  |
| *fepE* | AE014075 | Ferric enterobactin transport protein |  |  |  |  |  |
| *fepG* | AE014075 | Subunit of a ferric enterobactin ABC transporter complex |  |  |  |  |  |
| *fes* | CP027060 | Enterochelin esterase - upon internalization, ferric enterobactin is processed via an exquisitely specific pathway that is dependent on FES activity, making iron available for metabolic use |  |  |  |  |  |
| *fiu* | CP027060 | Involved in the active transport across the outer membrane of iron complexed with catecholate siderophores such as dihydroxybenzoylserine and dihydroxybenzoate |  |  |  |  |  |
| *flgD* | CP027060 | Deletion of *flgD* attenuated ExPEC strain PCN033 invasion and colonization in vivo, probably by affecting bacterial adhesion and invasion |  |  |  |  |  |
| *flgM* | CP028192 | Negative regulator of flagellin synthesis (Anti-sigma factor) |  |  |  |  |  |
| *fmlA* | BA000007 | F9/Yde/Fml pilus is involved in UPEC persistence in the inflamed urothelium |  |  |  |  |  |
| *fyuA* | CP016828 | The ferric yersiniabactin uptake receptor *fyuA* is required for efficient biofilm formation in UPEC |  |  |  |  |  |
| *gad* | FN554766 | Glutamate decarboxylase |  |  |  |  |  |
| *hlyD* |  | Hemolysin secretion protein |  |  |  |  |  |
| *hlyE* | BA000007 | Toxin, which has some hemolytic activity towards mammalian cells |  |  |  |  |  |
| *hlyIII* | CP003034 | inner membrane protein, hemolysin III family |  |  |  |  |  |
| *ibeB* | AF094824 | Important determinant contributing to *E. coli* K1 crossing of the blood–brain barrier |  |  |  |  |  |
| *ibeC* | CP019777 | Invasin of brain endothelial cells |  |  |  |  |  |
| *ibrA* | CP003034 | Immunoglobulin-binding regulator |  |  |  |  |  |
| *iha* | AF399919 | Virulence Factor in Murine Urinary Tract Infection |  |  |  |  |  |
| *invA* | CU928163 | May be a part of ETT2 |  |  |  |  |  |
| *invE* | NC_011751 | May be a part of ETT2 |  |  |  |  |  |
| *ipaH-like* | CU928164 | Invasion plasmid antigen of EIEC and Shigella |  |  |  |  |  |
| *irp1* | CU928163 | HMWP1 non-ribosomal peptide/polyketide synthase, part of HPI |  |  |  |  |  |
| *irp2* | CP006834 | HMWP2 Yersiniabactin biosynthetic protein, part of HPI |  |  |  |  |  |
| IS26 transposase | AP018456 | Plays a major role in the acquisition and dissemination of antibiotic resistance |  |  |  |  |  |
| *iss* | CP030791 | Increased serum survival |  |  |  |  |  |
| *iucA* | CU928163 | Part of the aerobactin gene cluster |  |  |  |  |  |
| *iucB* | AE014075 | Part of the aerobactin gene cluster |  |  |  |  |  |
| *iucC* | AE014075 | Part of the aerobactin gene cluster |  |  |  |  |  |
| *iucD* | CP001232 | Part of the aerobactin gene cluster |  |  |  |  |  |
| *iutA* | CP011134 | Ferric aerobactin receptor |  |  |  |  |  |
| *kpsM* | CU928163 | The deletion of gene *kpsM* weakens the virulence of porcine ExPEC PCN033 |  |  |  |  |  |
| *kpsMII* | X53819 | Associated with persistence or relapse in recurrent urinary tract infections caused by *E. coli* |  |  |  |  |  |
| *kpsS* | CU928163 | Capsule polysaccharide export protein |  |  |  |  |  |
| *lpfA* | CP006834 | Long polar fimbriae (LPF) are related to type I fimbriae |  |  |  |  |  |
| *lpfA-O113* | AY057066 | Long polar fimbriae (LPF) are related to type I fimbriae |  |  |  |  |  |
| *matD* | HM102365 | Meningitis-associated and temperature-regulated (Mat) fimbrial gene |  |  |  |  |  |
| *nfaA/dafaA* | AF325672 | Diffuse adherence fibrillar adhesin gene locus. Non-fimbrial adhesin 1. The *nfaA* gene encoding the antigen adhesive factor of enterotoxigenic *E. coli*. |  |  |  |  |  |
| *nfaB/dafaB* | AF325672 | Diffuse adherence fibrillar adhesin |  |  |  |  |  |
| *ompA* | CP027060 | Required for the action of colicins K and L and for the stabilization of mating aggregates in conjugation. |  |  |  |  |  |
| *ompW* | CP027060 | Receptor for Colicin S4 |  |  |  |  |  |
| *pap* operon | AP018784/ CP003034 | *pap* (pyelonephritis-associated pili) operon associated with UPEC strains. |  |  |  |  |  |
| *pgaA* | NZ_UGAE01000003 | Biofilm related gene |  |  |  |  |  |
| *ppk* | CP025268 | In some mutants lacking *ppk*, the phenotypes included features indicative of decreased virulence such as: (i) growth defects, (ii) defective responses to stress and starvation, (iii) loss of viability, (iv) polymyxin sensitivity, (v) intolerance to acid and heat, and (vi) diminished invasiveness in epithelial cells |  |  |  |  |  |
| *rafA* | NC_010558 | Peripheral raffinose metabolic pathway |  |  |  |  |  |
| *rafB* | NC_010558 | Peripheral raffinose metabolic pathway |  |  |  |  |  |
| *rafD* | NC_010558 | Peripheral raffinose metabolic pathway |  |  |  |  |  |
| *sat* | CU928163 | The secreted autotransporter toxin of UPEC |  |  |  |  |  |
| *senB* | AP018458 | May have some role in enterotoxicity of EIEC |  |  |  |  |  |
| *sfmC* | NSBV01000011 | Could contribute to adhesion to various surfaces in specific environmental niches. Increases adhesion to eukaryotic T24 bladder epithelial cells in the absence of *fim* genes |  |  |  |  |  |
| *shET2* | NZ_AYOG01000052 | Enterotoxin |  |  |  |  |  |
| *sinH* | CP023644 | Recent work suggests that at least two other virulence-associated bacterial outer membrane proteins share a structural and evolutionary history with intimin and invasin |  |  |  |  |  |
| *sitA* | FQ482074 | Homolog of the iron transport system SitABCD encoded on SPI1, which is required for full virulence of Salmonella typhimurium |  |  |  |  |  |
| *sitB* | NC_017659 | Homolog of the iron transport system SitABCD encoded on SPI1, which is required for full virulence of Salmonella typhimurium |  |  |  |  |  |
| *sitC* | CP000836 | Homolog of the iron transport system SitABCD encoded on SPI1, which is required for full virulence of Salmonella typhimurium |  |  |  |  |  |
| *sitD* | NC_017659 | Homolog of the iron transport system SitABCD encoded on SPI1, which is required for full virulence of Salmonella typhimurium |  |  |  |  |  |
| *stcD* | NC_018658 | Fimbrial-like adhesin protein |  |  |  |  |  |
| *tia* | DQ095216 | Invasion determinant |  |  |  |  |  |
| *traJ* | DQ381420 | Contributes to *E. coli* K1 virulence in the neonatal rat |  |  |  |  |  |
| *traT* | AY214164 | May inhibit the formation of C5b6 complex or causes structural alteration of the complex to a non-functional form |  |  |  |  |  |
| *upaG-like* |  | Autotransporter protein of UPEC |  |  |  |  |  |
| *ybtA* | CP028714 | Yersiniabactin transcriptional regulator |  |  |  |  |  |
| *ybtQ* | CP003034 | Yersiniabactin-iron ABC transporter permease |  |  |  |  |  |
| *ybtX* | CP003034 | Yersiniabactin-iron transporter permease |  |  |  |  |  |
| *ycgV* | NC_011751 | Putative outer membrane autotransporter/Predicted adhesin. The YcgV-encoding gene was found in the majority of commensal and DEC genomes (it was truncated in both EHEC strains); however, the entire gene was missing from every single UPEC genome |  |  |  |  |  |
| *ydeR* | CU928163 | Fimbrial protein that is significantly phylogroup B1-associated |  |  |  |  |  |
| *ygeF* | NC_002695 | ETT2 gene |  |  |  |  |  |
| *ygeG* | NC_002695 | ETT2 gene |  |  |  |  |  |
| *ygeH* | NC_002695 | EilA and YgeH proteins show a moderate similarity to HilA and are encoded in pathogenicity islands from several *E. coli* strains, both pathogenic and non-pathogenic |  |  |  |  |  |
| *ygeI* | NC_011750 | ETT2 gene |  |  |  |  |  |
| *yghJ* | KX245009 | Secreted metalloprotease of pathogenic *E. coli* induces hemorrhagic fluid accumulation in mouse ileal loop |  |  |  |  |  |
| *ypjA* | NC_011751 | Identified as a protein with similarity to antigen 43 (Ag43), a self-recognizing surface adhesin |  |  |  |  |  |
| *yqfA* | CP027060 | Hemolysin III family protein |  |  |  |  |  |
| *yqgB* | CP023258 | Acid stress response protein |  |  |  |  |  |
